# The Current Knowledge of the Role of PPAR in Hepatic Ischemia-Reperfusion Injury

**DOI:** 10.1155/2012/802384

**Published:** 2012-05-16

**Authors:** M. Elias-Miró, M. B. Jiménez-Castro, M. Mendes-Braz, A. Casillas-Ramírez, C. Peralta

**Affiliations:** ^1^Institut d'Investigacions Biomèdiques August Pi i Sunyer (IDIBAPS), Esther Koplowitz Center, Roselló 149–153, 3rd Floor, Office 3.8, 08036 Barcelona, Spain; ^2^Departamento de Patologia e Medicina Legal, Faculdade de Medicina, Universidade de Sao Paulo, 14049-900 Sao Paulo, SP, Brazil; ^3^Centro de Investigación Biomédica en Red de Enfermedades Hepáticas y Digestivas, 08036 Barcelona, Spain

## Abstract

Strategies to improve the viability of steatotic livers could reduce the risk of dysfunction after surgery and increase the number of organs suitable for transplantation. Peroxisome proliferator-activated receptors (PPARs) are major regulators of lipid metabolism and inflammation. In this paper, we review the PPAR signaling pathways and present some of their lesser-known functions in liver regeneration. Potential therapies based on PPAR regulation will be discussed. The data suggest that further investigations are required to elucidate whether PPAR could be a potential therapeutic target in liver surgery and to determine the most effective therapies that selectively regulate PPAR with minor side effects.

## 1. Introduction

Liver transplantation has evolved as the therapy of choice for patients with end-stage liver disease. However, the waiting list for liver transplantation is growing at a rapid pace, whereas the number of available organs is not increasing proportionately. The potential use of steatotic livers, one of the most common types of organs in marginal donors, for transplantation has become a major focus of investigation. However, steatotic livers are more susceptible to ischemia-reperfusion (I/R) injury, and the transplantation of steatotic levels results in a poorer outcome than that of nonsteatotic livers. Indeed, the use of steatotic livers for transplantation is associated with an increased risk of primary nonfunction or dysfunction after surgery [1, 2]. In hepatic resections, the operative mortality associated with steatosis exceeds 14%, compared with 2% for healthy livers, and the risks of dysfunction after surgery are similarly higher [2, 3]. Despite advances aimed at reducing the incidence of hepatic I/R injury (summarized in earlier reviews) [1, 2], the results to date are inconclusive. In this paper, we review the peroxisome proliferator-activated receptor alpha (PPAR*α*) and PPAR*γ* signaling pathways in steatosis, inflammation and regeneration, three key factors in steatotic liver surgery [1–5]. Our review of the different strategies pursued to regulate PPAR in liver diseases may motivate researchers to develop effective treatments for steatotic livers in patients undergoing I/R. The potential clinical application of strategies that regulate PPAR in the setting of steatotic liver surgery is also discussed.

## 2. Characteristics of PPAR

PPARs belong to the hormone nuclear receptor superfamily and consist of three isoforms: PPAR*α*, PPAR*γ*, and PPAR*β*/*δ*. Of these, our group and others have demonstrated that PPAR*α* and PPAR*γ* are important regulators of postischemic liver injury [1, 2, 6, 7] that exert their effects on steatosis and inflammation, which is inherent in steatotic liver surgery [8–12].

Previous results indicate that the presence of fatty infiltration by itself in the liver (without any surgical intervention) does not induce changes in PPAR*α* or PPAR*γ* levels, as no differences were observed in the levels of these transcription factors between steatotic and nonsteatotic livers of a sham group of Zucker rats [13, 14]. These results contrast reports from the literature indicating high or low PPAR*γ* levels in steatotic livers compared with those in nonsteatotic livers [15, 16]. These different results can be explained, at least in part, by differences in the level of PPAR*γ* regulation between rats and mice [17], the different obesity experimental models evaluated, and the degree of steatosis. We reported that PPAR*γ* expression levels in nonsteatotic livers during liver transplantation were similar to those observed in the sham group. However, increased PPAR*γ* levels were observed in steatotic liver grafts [14, 18]. Thus, steatotic liver grafts are more predisposed to overexpress PPAR*γ*. This is in line with clinical studies, in which PPAR*γ* was upregulated in the livers of obese patients with nonalcoholic fatty liver disease (NALFD) [19]. Additionally, differences in PPAR*α* expression were observed among different liver types. Indeed, steatotic livers are more predisposed to downregulate PPAR*α*, when they are subjected to warm hepatic ischemia [13]. In line with these findings, PPAR*α* is downregulated in the livers of obese patients with NALFD [20]. Findings such as these must be considered when applying the same pharmacological strategies indiscriminately to patients with steatotic and nonsteatotic livers because the effects may be very different.

**Figure 1:**
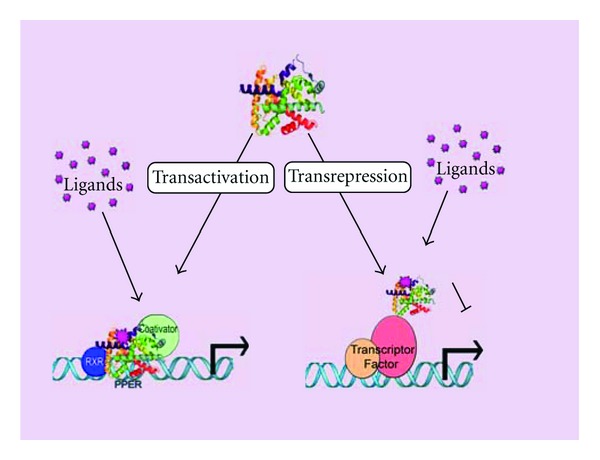
Basic mechanism of PPAR action. Receptor X retinoide, RXR; PPAR-response element, PPER.

PPARs can both activate and inhibit gene expression by two mechanisms: transactivation and transrepression. Transactivation is DNA- and ligand-dependent. PPARs activate transcription in a ligand-dependent manner by binding directly to specific PPAR response elements (PPREs) in target genes as heterodimers with retinoid X receptor (RXR). Agonist binding leads to the recruitment of coactivator complexes that modify the structure of chromatin and facilitate the assembly of the general transcriptional machinery at the promoter [21]. Transrepression is ligand-dependent and may explain the anti-inflammatory actions of PPARs [22]. PPARs repress transcription by antagonizing the actions of other transcription factors [21] (see Figure 1). Physiologically, PPAR-RXR heterodimers may bind to PPREs in the absence of a ligand. Although the transcriptional activation depends on the ligand-bound PPAR-RXR, the presence of unliganded PPAR-RXR at a PPRE has effects that vary depending on the promoter context and cell type [22]. Further investigations on the structures of PPARs and the mechanisms by which PPARs regulate gene transcription may be useful for designing certain strategies, such as the use of PPAR antagonists or agonists. As shown in the following sections, the currently used pharmacological strategies aimed at regulating PPAR could not be incorporated into liver surgery due to their potential side effects.

Given the antiobesity and anti-inflammatory properties of PPAR*α* and PPAR*γ* [8–12], pharmacological interventions targeting these transcription factors could be a promising strategy to treat hepatic steatosis in patients undergoing I/R. However, as shown in Figure 1, the effects of pharmacological strategies aimed at modulating PPARs depend on the type of ischemia (cold or warm ischemia), the length of ischemia, and the type of the liver (nonsteatotic or steatotic liver). 

## 3. Effect of PPAR on Hepatic I/R

**Figure 2:**
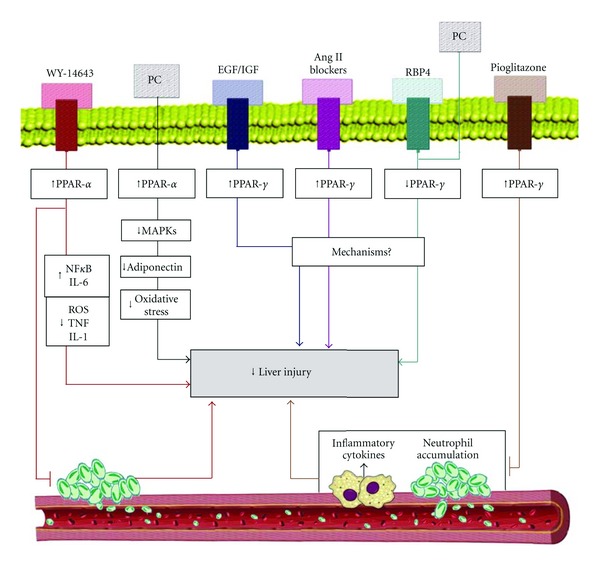
PPAR and hepatic I/R injury. Angiotensin II, Ang II; epidermal growth factor, EGF; insulin-like growth factor, IGF; interleukin-6, IL-6; mitogen-activated protein kinases, MAPKs; nuclear factor kappa B, NF*κ*B; PPAR*α* agonist; pioglitazone, peroxisome proliferator-activated receptors, PPAR; ischemic preconditioning, PC; retinol binding protein, RBP4, PPAR*α* agonist; Wy-14,643.

To the best of our knowledge, few studies have examined both the I/R-inducedexpression of hepatic PPAR*α* and the potential benefits of PPAR*α* agonists under these conditions. According to previous studies by our group, PPAR*α* mRNA and protein levels in nonsteatotic livers during I/R were similar to those of the sham group, and PPAR*α* did not play a crucial role in I/R injury in nonsteatotic livers [13]. This contrasts studies published by Okaya and Lentsch [23] and Xu et al. [24], who reported the benefits of PPAR*α* agonists in postischemic liver injury. The protective effects were possibly associated with reductions in neutrophil accumulation, oxidative stress, and tumor necrosis factor (TNF) and interleukin-1 (IL-1) expression (Figure 2). Although the dose and pretreatment time of the PPAR*α* agonist WY-14,643 were similar in both studies, Okaya and Lentsch [23] and Xu et al. [24], reported an ischemic period of 90 min [23, 24]; our ischemic period was 60 min, which is the ischemic period currently used in liver surgery [13]. Thus, 60 min of ischemia appears insufficient for inducing changes in PPAR*α* levels in nonsteatotic livers. In nonalcoholic steatohepatitis (NASH) and simple steatosis, treatment of mice with the PPAR activator Wy-14,643 protects steatotic livers against I/R injury, and the benefits of this treatment potentially occur through the dampening of adhesion molecule and cytokine responses and activation of nuclear factor kappa B (NF-*κ*B) and IL-6 production [25]. In steatotic livers undergoing warm ischemia, PPAR*α* agonists can limit the damage induced by I/R. PPAR*α* agonists as well as ischemic preconditioning (PC) through PPAR*α* inhibited mitogen-activated protein kinases (MAPK) expression following I/R (Figure 2). This in turn inhibited adiponectin accumulation in steatotic livers and adiponectin worsening effects on oxidative stress and hepatic injury [13]. Given these data, PPAR*α* regulation could be an alternative method for reducing the greater oxidative stress incurred by steatotic livers. Indeed, preventing I/R injury in steatotic livers via therapies aimed at inhibiting reactive oxygen species (ROS) production has proven difficult. Steatotic livers might produce SOD/catalase-insensitive ROS, which may be involved in the mechanism of failure of steatotic livers after transplantation [26]. Moreover, gene therapy based on antioxidant overexpression is limited by the toxicity of the vectors [2, 27]. In a recent study of nonsteatotic livers undergoing warm hepatic ischemia, the dietary supplementation with n-3  polyunsaturated fatty acids (PUFAs) increased hepatic  n-3 PUFA content and reduced hepatic n-6/n-3 PUFA content. This was associated with PPAR*α* upregulation, which in turn reduced NF-*κ*B signaling and oxidative stress, leading to a reduced inflammatory response [28].

The function of PPAR*γ* in hepatic I/R injury is unclear. Previous results in liver transplantation studies indicated that I/R did not induce changes in PPAR*γ* expression in nonsteatotic livers, and consequently, strategies based on PPAR*γ* regulation had no effect on hepatic injury [14]. These results were different from those observed in nonsteatotic livers under warm ischemia conditions [6]. In that study, treatment with pioglitazone, a PPAR*γ* agonist, significantly inhibited hepatic I/R injury (Figure 2). The protective effect was associated with the downregulation of several proinflammatory cytokines and chemokines and neutrophil accumulation [7]. This is in line with other results indicating that PPAR*γ*-deficient mice displayed more severe injuries than untreated mice under warm ischemia conditions [6]. Furthermore, pioglitazone treatment inhibited apoptosis and significantly improved the survival of mice in a lethal model of hepatic I/R injury [7]. Previous studies indicated that PPAR*γ* activation inhibits the release of TNF*α*, IL-1, and IL-6 by macrophages [29, 30], which could be of interest in steatotic livers. Indeed, under warm hepatic ischemia, higher IL-1 and lower IL-10 levels were detected in steatotic livers after reperfusion than in nonsteatotic livers [31]. This imbalance between pro- and anti-inflammatory ILs increased oxidative stress and decreased the tolerance of steatotic livers to I/R. In addition, different studies have reported proinflammatory and anti-inflammatory roles of TNF-*α* and IL-6, respectively, in the vulnerability of steatotic livers undergoing I/R [2, 32].

Previous results indicated that PPAR*γ* activation in hepatocytes by rosiglitazone treatment increases autophagy and protects against hepatic I/R injury. Autophagy is an evolutionarily conserved cellular process for recycling of old proteins and organelles via the lysosomal degradation [33]. Thus, these results suggest that PPAR*γ* has anti-inflammatory properties and therefore may be relevant during hepatic I/R injury. In line with these data, PPAR*γ* upregulation is a key mechanism of the benefits of different pharmacological or surgical strategies for steatotic livers undergoing I/R. Thus, some results based on isolated perfused livers indicated that the addition of growth factors (epidermal growth factor (EGF) and insulin-like growth factor-1 (IGF-I)) to University of Wisconsin (UW) preservation solution protected steatotic livers due to PPAR*γ* overexpression [34]. Similarly, EGF pretreatment mediated by PPAR*γ* overexpression protected steatotic livers undergoing warm ischemia [35] (Figure 2). Moreover, in warm hepatic ischemia, PPAR*γ* upregulation was a key mechanism of the benefits of pharmacological blockers of angiotensin II (angiotensin-converting-enzyme (ACE) inhibitors and Ang II receptor antagonists) on steatotic livers [36]. However, the role of PPAR*γ* in hepatic I/R injury could depend on the surgical conditions, as a recent study of liver transplantation indicated that treatment with a PPAR*γ* antagonist was effective in steatotic livers, suggesting a detrimental role of PPAR*γ* under these conditions [14]. In line with this finding, PPAR*γ* inhibition was a key mechanism of the benefits of RBP4 treatment and PC on steatotic liver grafts [14]. Considering these results, drugs targeting PPAR*γ* regulation can potentially increase the number of organs suitable for transplantation, as these drugs can improve the outcome for marginal grafts that would not otherwise have been transplanted. However, the data on PPAR*γ* reported in steatotic liver transplantation models with standard liver graft sizes should not be extrapolated to small-size steatotic liver grafts. In the case of small liver transplants, the liver regeneration inherent in this surgical procedure and the mechanism of hepatic damage derived from the removal of hepatic mass should be considered [1, 31, 36]. In small liver grafts the periods of ischemia ranged 40–60 min, whereas the periods of ischemia ranged 6–8 hours for cadaveric donor liver transplantation.

## 4. Effect of PPAR on Hepatic Steatosis

Numerous studies suggest that the actions of PPAR*α* can prevent steatosis. Mice deficient in PPAR*α* develop hepatic steatosis when fasted or fed a high-fat diet [37, 46, 57]. Treatment with a PPAR*α* agonist decreased hepatic steatosis in mice on a methionine- and choline-deficient (MCD) diet [37]. Activation of PPAR*α* by the agonist Wy-14,643 ameliorated alcoholic fatty liver- and MCD-induced steatohepatitis [37, 38]. The critical role of PPAR*α* in ameliorating steatosis is mediated through the regulation of a wide variety of genes involved in peroxisomal, mitochondrial, and microsomal FA *β*-oxidation systems in the liver [58]. When steatotic livers are submitted to certain stresses much as partial hepatectomy, the activation of PPAR*α* by bezafibrate reduces the availability of FAs from circulation, reducing thus the hepatic sphingolipid synthesis [40] (see Table 1).

It is well known that n-3 PUFAs and their derivative FAs activate PPAR*α* [59–61], which then heterodimerizes with RXR and liver X receptor, leading to the transcription of a large number of genes involved in lipid metabolism. It has been reported that n-3 PUFAs are more potent than the n-6  PUFAs as in vivo activators of PPAR*α* [59]. In addition, PUFA metabolites such as eicosanoids or oxidized FAs have one to two orders of magnitude greater affinity for PPAR*α* and are consequently far more potent transcriptional activators of PPAR*α*-dependent genes [59].

The interaction of PPAR*α* with its DNA recognition site is markedly enhanced by ligands such as hypotriglyceridemic fibrate drugs, conjugated linoleic acid, and PUFAs [59]. The discovery of PPAR*α* led quickly to the idea that PPAR*α* was a “master switch” transcription factor that was targeted by PUFA to coordinately suppress genes encoding lipid synthesis proteins and to induce genes encoding lipid oxidation proteins [59]. In line with this idea, recent studies suggested that n-3 FAs serve as important mediators of gene expression, working via the PPARs to control the expression of the genes involved in lipid and glucose metabolism and adipogenesis [61]. Neschen et al. [62] demostrated that the administration of dietary fish oil (n-3) to rats increases the FA capacity of their livers through its ability to function as a ligand activator of PPAR*α* and thereby induces the transcription of several gene-encoding proteins affiliated with FA oxidation. Of interest, other studies examining the effects of fish oil feeding on the expression of several genes of PPAR knockout mice clearly indicated that hepatic gene regulation by fish oil feeding involves at least two different pathways: PPAR*α*-dependent and PPAR*α*-independent pathways. Enzymes for peroxisomal (CYP4A2) and microsomal (AOX) oxidation are PPAR*α*-dependent and upregulated by fish oil feeding, whereas those for lipid synthesis (FAS; S14) are PPAR*α*-independent and downregulated. This indicates that the FA regulation of de novo hepatic lipogenesis and FA oxidation are not mediated through a common factor (e.g., PPAR*α*) [61].

Given all these data into in account, the regulation of PPAR*α* by PUFA, particularly n-3 PUFA and possibly conjugated linoleic acid, may offer an explanation for the reported benefits of these FAs in different pathologies.

In obese NAFLD patients, the increased production of ROS leads to the depletion of n-3 PUFAs due to enhanced lipid peroxidation. As PPAR*α* is activated through direct binding to n-3 PUFA, liver PPAR*α* function is compromised in obesity. This prevented the upregulation of genes involved in lipid transport, FA *β*-oxidation and thermogenesis, favoring FA and triacylglycerol synthesis over FA *β*-oxidation and thus promoting hepatic steatosis [20]. Thus, PPAR*α* activation by n-3 PUFA supplementation ameliorated hepatic steatosis in obese NAFLD patients [20]. In line with this, NASH patients have low levels of circulating n-3 PUFA, with a consequent increase of the n-6/n-3 FA ratio and impaired PPAR*α* activity in the liver [42, 43]. NASH patients treated with eicosapentaenoic acid (EPA) or n-3 PUFAs, a mixture of EPA and docosahexaenoic acid, exhibited improvements in hepatic steatosis and necroinflammation in humans and rats with NASH, probably due to the reduction of hepatic TNF*α* expression and improvement of insulin sensitivity [41–43]. Moreover, PUFAs activate PPAR*α*, leading to increased FA *β*-oxidation; hence, they can shift the energy balance from storage to consumption [41, 43].  n-3 PUFAs have also been proved as safe and efficacious for patients with NAFLD associated with hyperlipidemia, as indicated by reduced hepatic damage and serum lipid levels [44]. In another study, the efficacy and safety of three hypolipidemic, agents in patients with NAFLD with dyslipidemia were evaluated. In this context, predominantly hypertriglyceridemic, hypercholesterolemic, and overweight patients were treated with n-3 FAs, atorvastatin, and orlistat, respectively. The three different groups of patients exhibited reduced hepatic damage, normalized of hepatic steatosis, and reduced serum lipids [45].

Considering that steatosis is a risk factor in liver surgery, strategies aimed to reduce steatosis could increase the tolerance of steatotic livers to I/R. There is considerable evidence that liver regeneration is impaired in certain genetic models in which the liver contains excess fat. For example, steatotic livers from Ob mice exhibit defective liver regeneration and high mortality following partial hepatectomy [63]. Similarly, impaired liver regeneration was observed in steatotic livers undergoing partial hepatectomy under vascular occlusion compared with that in nonsteatotic livers [31]. On the contrary, drugs that reduce hepatic steatosis, such as PPAR*α* regulators, should be considered with caution in clinical liver surgery, as other studies indicate that genetic or pharmacologic approaches that reduce lipid accumulation may also hinder liver regeneration [63–66]. Thus, a question is to what degree should we reduce steatosis in steatotic livers to protect this type of liver. Another question is whether we should reduce steatosis before the surgical procedure and therefore avoid the vulnerability of steatotic livers to I/R, or in contrast, should we use drugs aimed at reducing hepatic triglycerides during surgery and thus conserve the energy required for liver regeneration. Moreover, research evaluating whether the short-term administration of PPAR*α* agonists might alleviate hepatic steatosis in steatotic livers before I/R would be of interest for clinical practice because there are obvious difficulties concerning the feasibility of long-term PPAR*α* agonist administration in some I/R processes, in particular liver transplantation from cadaveric donors, because this is an emergency procedure in which there is very little time to pretreat the donor with PPAR*α* agonists.

Several studies attribute a causal role to PPAR*γ* in the development of steatosis by mechanisms involving the activation of lipogenic genes and de novo lipogenesis [48, 51]. In accordance, targeted deletion of PPAR*γ* in hepatocytes protects mice against diet-induced hepatic steatosis [67], suggesting a prosteatotic role of PPAR*γ*. Similarly, mice with liver-specific PPAR*γ* silencing are protected against hepatic steatosis [56]. Additionally, treatment of ob/ob mice with rosiglitazone increased liver steatosis [49]. By contrast, different results have been reported regarding the effect of PPAR*γ* on hepatic steatosis. Indeed, PPAR*γ*-deficient mice develop more severe MCD-induced NAFLD, whereas adenovirus-mediated PPAR*γ* overexpression attenuated the progression of NASH [55]. In line with this finding, rosiglitazone treatment prevented the development of NASH in a model of MCD-treated mice [55], and similar results were obtained using the PPAR*γ* agonist pioglitazone [52, 53]. These different results can be partially explained by differences in the studies such as the species, type of PPAR agonist, method to induce hepatic steatosis, the type of genetic strategy used to induce PPAR*γ* overexpression or deficiency in PPAR*γ* expression as well as differences in the pretreatment times of the drugs used (see Table 1).

## 5. Effect of PPAR on Hepatic Regeneration

**Figure 3:**
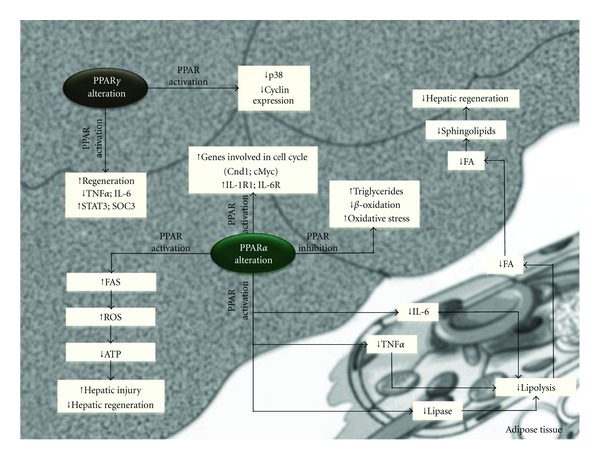
PPAR and hepatic regeneration. Adenosine triphosphate: ATP; fatty acid: FA; interleukin-1 receptor: IL-1R; interleukin-6: IL-6; interleukin-6 receptor: IL-6R; tumor necrosis factor-alpha: TNF-*α*; signal transducer and activator of transcription 3: STAT3; suppressor of cytokine signalling 3: SOC3; reactive oxygen species: ROS.

Recent studies demostrated that liver regeneration is impaired in a number of animal models of fatty liver disease [68–73]. PPAR*α*-null mice subjected to partial hepatectomy (PH) have an impaired ability to regenerate hepatic mass. Emerging evidence suggests that PPAR*α* is a critical modulator of the energy flux important for the repair of liver damage. For example, hepatocytes in the periportal regions, which divide and replicate after PH, require mitochondrial oxidation of FAs to generate energy [74]. PPAR*α* controls the constitutive expression of genes involved in mitochondrial FA oxidation, including carnitine palmitoyltransferase-1 [46, 75]. In mice deficient in PPAR*α*, the impaired hepatic regeneration is also associated with the altered expression of genes involved in cell cycle control and cytokine signaling. Studies with PPAR*α* agonists indicate that PPAR*α* upregulates genes involved in the cycle cell (Ccnd1 and cMyc) as well as IL1r1 and IL-6r [76] (Figure 3).

It is well known that PPAR*α* affects the transcription of a number of genes involved in lipid turnover and peroxisomal and mitochondrial *β*-oxidation, resulting in the generation of ATP, which is required to “fuel” liver repair and regeneration [76]. By contrast, in conditions in which PPAR*α* function and/or expression is altered such as hepatic steatosis, and small-size liver grafts, FA metabolism is deviated toward the accumulation of inadequately metabolized fat, favoring ROS generation. Consequently, ATP production is decreased, and the demise of hepatocytes via necrotic cell death is increased, halting liver repair [77] (Figure 3). Accordingly, mice with targeted PPAR*α* disruption exhibit increased inflammation and necrosis and delayed liver regeneration following partial hepatectomy [47].

Previous results indicate that the impaired liver regeneration of steatotic rats was partially due to PPAR*α* downregulation through the AdipoR2 axis. The inhibition of PPAR*α* signaling, increased triglyceride (TG) accumulation in hepatocytes and inhibited the expression of hepatic enzymes that contribute to FA oxidation (Figure 3). This was associated with increased lipid peroxidation and decreased antioxidant levels [78].

In contrast with the aforementioned data indicating the beneficial effects of PPAR*α* on hepatic regeneration, a recent report indicated that PPAR*α* activation by bezafibrate had negative effects on liver regeneration, which can be attributed to the inhibition of de novo sphingolipid synthesis [40]. Presumably, bezafibrate affects de novo sphingolipid synthesis by decreasing FA availability (Figure 3). The activation of PPAR*α* by bezafibrate virtually obliterated the postoperative increase in plasma nonesterified FAs induced by PH. This can be explained by the inhibition of hormone-sensitive lipase activity in adipose tissue by PPAR*α* ligands and their anti-inflammatory properties, which decrease the release of cytokines such as TNF and IL-6. Both events inhibited lipolysis in isolated white adipocytes, resulting in reduced FA release from extrahepatic sources after PH [40].

PPAR*γ* activity is likely to be regulated during normal liver regeneration, and the disruption of this regulation could impair the regenerative response. Pioglitazone improved hepatic regeneration failure in obese mice. This effect was associated with reduced TNF*α* and IL-6 levels. Additionally, pioglitazone prevented the increased mRNA expression of signal transducer and activators of transcription-3 phosphorylation and suppressor of cytokine signaling-3 mRNA in the livers of obese mice [54]. However, inconsistent results have been obtained regarding the effect of PPAR*γ* of liver regeneration. Indeed, rosiglitazone inhibited hepatocyte proliferation in mice undergoing partial hepatectomy by reducing p38 and cyclin expression [50] (see Figure 3).

On the basis of the inconsistent results reported to date on the role of PPAR in hepatic regeneration, it is difficult to discern whether we should attempt to inhibit PPAR or administer PPAR activators to promote liver regeneration in surgery.

## 6. Modulators of PPAR in Clinical Practice

Based on the data reported in experimental models (as reviewed above), different strategies (which have been summarized in Table 1) could exert effects on steatosis, inflammation, or regeneration by regulating PPAR. Whether these pharmacological approaches can be translated into treatments for clinical liver surgery remains unknown. For example, thiazolidinediones (TZDs) should not be applied in clinical liver surgery due to their potential side effects. TZDs (pioglitazone, troglitazone, and rosiglitazone) are synthetic PPAR*γ* agonists that are widely used as antidiabetic agents [79–81]. However, prolonged treatment of obese and diabetic mice with TZDs resulted in the development of severe steatosis, which can lead to steatohepatitis and/or fibrosis. Troglitazone administration was associated with the development of idiosyncratic acute liver failure and was therefore withdrawn from clinical use [82, 83]. Hepatotoxicity has subsequently been reported in patients taking pioglitazone and rosiglitazone [83, 84]. These data provide support for current clinical practices in which these drugs are avoided or used judiciously in patients with known or suspected liver disease. Further experiments should be initiated to devise a pharmaceutical form appropriate for clinical use.

**Figure 4:**
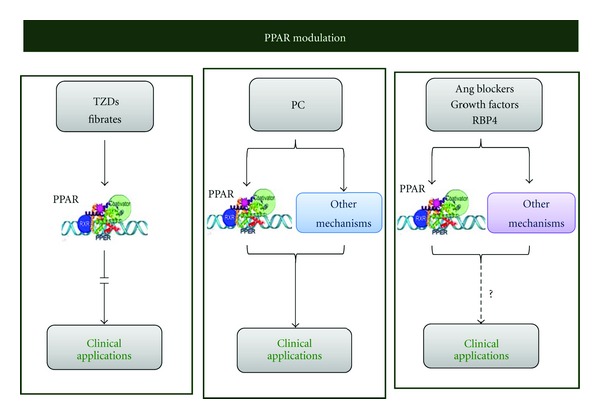
Clinical application of strategies that regulate PPARs. Angiotensin: Ang; peroxisome proliferator-activated receptors: PPARs; ischemic preconditioning: PC; retinol binding protein: RBP4; thiazolidinediones: TZDs.

PPAR*α* agonists are clinically and functionally relevant as fibrate therapeutics against hyperlipidemia and agents for reducing the complications of peripheral vascular disease in diabetic patients [85]. Despite their potentially beneficial roles, PPAR*α* agonists should be used judiciously. Short-term administration in humans (1–10 days) would be unlikely to produce permanent genotoxic effects. However, long-term exposure to these drugs, which would be required to reduce hepatic steatosis, can result in oxidative DNA damage, among other effects [86–90] (Figure 4).

Further studies will also be required to elucidate whether growth factors, Ang II blockers, or RBP4 may be safer protective pharmacologic strategies for regulating PPAR in hepatic I/R injury in clinical practice (Figure 4). Nevertheless, none of the aforementioned strategies is specific for PPAR.

To avoid the potential side effects of PPAR agonists, strategies that regulate PPAR*α*, such as the induction of PC could be of clinical interest. PC is an adaptive mechanism that consists of a brief period of I/R, resulting in marked resistance in the liver, prior to a subsequent prolonged ischemic stress. Our successes regarding the efficacy of PC in nonsteatotic and steatotic livers undergoing warm ischemia (associated with PH) and liver transplantation [1, 2, 14, 91–93] have resulted in the clinical application of PC.

Several studies have demonstrated the effectiveness of PC in the resection of steatotic and nonsteatotic livers in clinical practice [94–96]. In such studies, the authors primarily performed liver resection via a continuous Pringle maneuver. However, other data indicate that PC does not improve postoperative liver function and does not affect morbidity or mortality after hepatectomy under vascular exclusion of the liver with the preservation of caval flow [97, 98]. The discrepancy between these differential effects of PC during hepatic resection might have arisen from the absence of back flow perfusion of the liver during vascular exclusion compared with that during the Pringle maneuver, which involves interruptions only to the inflow to the liver. In addition, the ischemic period used by Azoulay et al. [97] was longer (10 min on average) that that used by Clavien et al. [94]. All of these could explain, at least partially, the different effectiveness of PC in the clinical practice of liver surgery.

In the past decade, serious efforts have commenced to translate some of the robust benefits of PC against ischemia reperfusion to liver transplantation in clinical practice. It is fair to conclude that the overall clinical results have been less impressive than the observations in experimental animals. There are different data on the effectiveness of PC in I/R injury associated with liver transplantation [99–102]. However, these differential effects cannot be explained by the use of PC periods that have proved experimentally ineffective or by the clinical use of different cold ischemic times from those evaluated experimentally. However, the reduced proportion of subjects with steatosis enrolled in PC trials and the presence of brain death in clinical liver transplantation, which has thus far been evaluated in experimental studies of liver transplantation, should be considered.

As previously mentioned, the proportion of subjects with steatosis who have been enrolled in PC trials to date has been small (10%). Thus, in the future, clinical trials must make serious efforts to include a larger proportion of donor with steatotic livers to clarify the effectiveness of PC in liver transplantation in clinical practice. The benefits of PC are more likely to become clinically meaningful in patient groups with an increased risk of morbidity and mortality following PH, that is, in patients with hepatic steatosis and cirrhosis. In fact, in the largest prospective randomized study of PC in PH, Clavien et al. [94, 103] demostrated that PC was more effective in reducing reperfusion injury in patients with steatotic livers. Furthermore, Li et al. [104] reported that PC decreased the risk of hepatic insufficiency and shortened the hospital stay in patients with cirrhosis who underwent PH. There is the remote possibility that PC may not be effective in the context of brain death. Deceased organ donors have hemodynamic instability with decreased mean arterial pressure, portal venous, and hepatic tissue blood flow. Furthermore, brain death induces a multifaceted, intense systemic inflammatory response that is manifested in many organs, including the liver. It is very likely that such a framework of inflammatory response, well entrenched before the induction of PC, would interact with the various mechanistic aspects of PC and modulate the eventual PC response. To our knowledge, there are no studies of PC in the livers in brain-dead animals. Additional experimental studies of PC of the liver and other organs in brain-dead animals are needed to fill the knowledge gaps. The clinical observations suggest that PC alone may be insufficient to provide easily demonstrable clinical benefits in the presence of brain death. In that context, PC may be more effective when combined with physical, chemical, and pharmacological PC methods. Such experimental investigations could address an important clinical problem in liver transplantation, as more than 80% of livers used for transplantation are taken from cadaveric donors and approximately 20% of all brain-dead donors have a mild-to-moderate hepatic steatosis [105].

## 7. Conclusions and Perspectives

The use of experimental models has contributed to a better understanding of the multifaceted roles of PPARs. Strategies based on PPAR regulation have the potential to improve the postoperative outcomes of patients undergoing hepatic resections and to increase the number of organs suitable for transplantation, as these strategies may improve the outcomes of patients receiving marginal grafts that would not otherwise have been transplanted, leading to new possibilities for small steatotic liver transplants. Before a complete definition of a successful therapeutic strategy based on PPAR regulation is formed, several additional points need to be addressed. Comparative studies of the roles of different PPAR isoforms in hepatic I/R are required. We recently mapped the effects of PPAR on the pathways involved in the inflammatory process and lipid metabolism, and the effects of PPAR differ according the experimental model used. Therefore, therapeutic strategies targeting PPAR regulation also differ according to the surgical procedure. Moreover, the response of different types of liver to PPAR stimulation might differ and involve different signal transduction pathways that are at present marginally understood. Further research is required to select drugs that regulate PPAR with minimal side effects and optimize such potential treatments (e.g., dose and pharmacokinetics) before being translated into treatments for human disease. Pharmacological strategies that specifically regulate PPAR including fibrates and TZDs might be inappropriate for clinical liver surgery due to their potential side effects. Conversely, surgical strategies such as PC have been applied in clinical surgery; however, these strategies do not exert their effects exclusively on PPAR, as they affect multiple aspects of I/R injury. Only a full appraisal of the role of PPAR in hepatic I/R and studies on the structure of this transcription factor will permit the design of new protective strategies for clinical liver surgery based on the specific regulation of PPAR without adverse effects.

## Figures and Tables

**Table 1:** Effect of strategies that regulate PPAR on hepatic injury, steatosis, and regeneration in experimental models and patients. Angiotensin II: Ang II; choline deficient: CD; epidermal growth factor: EGF; high-fat diet: HFD; insulin-like growth factor 1: IGF-1; methionine choline deficient: MCD; nonalcoholic Steatohepatitis: NASH; peroxisome proliferator-activated receptors: PPARs; polyunsaturated fatty acids: PUFAs; ischemic preconditioning: PC; retinol binding protein-4: RBP4.

PPAR*α*					
PPAR*α* activators					
Strategies	Time	Effect	Experimental model and patients	Steatosis and hepatic injury	Regeneration
WY-14,643 (30 *μ*mol/kg/d) [17]	3 weeks	↑ PPAR*α*	Obese Zucker rats	↑ *β*-oxidation of fatty acids	Not evaluated
WY-14,643 (180 *μ*mol/kg/d) [17]	1 week	↑ PPAR*α*	Ob/ob mice	↑ *β*-oxidation of fatty acids;↓ triglycerides	Not evaluated
WY-14,643 (10 mg/kg) [23, 24]	1 h before ischemia	↑ PPAR*α*	Mice or Rats; warm ischemia (90 min)	↓ hepatic injury	Not evaluated
WY-14,643 (10 mg/kg) [13]	1 h before ischemia	↑ PPAR*α*	Zucker obese rats; warm ischemia (60 min)	↓ hepatic injury	Not evaluated
WY-14,643 (10 mg/kg) [25]	10 days before surgery	↑ PPAR*α*	Foz/foz mice; steatotic livers; warm ischemia (90 min)	↓ hepatic injury	↑ cell cycle entry
Wy-14,643 (0.1%) [37]	5 weeks	↑ PPAR*α*	Mice fed MCD diet	↓ steatohepatitis	Not evaluated
Wy-14,643 (0.1%) [38]	12 days	↑ PPAR*α*	Mice fed MCD diet	↓ steatohepatitis; ↑ hepatic fatty acid oxidation	Not evaluated
Bezafibrate [39]	5 weeks	↑ PPAR*α*	Mice fed MCD	↓ hepatic triglycerides; ↑ hepatic fatty acid oxidation	Not evaluated
Benzafibrate (75 mg/kg) [40]	7 days	↑ PPAR*α*	Rats; partial hepatectomy	↓ availability of fatty acids; sphingolipid synthesis	↓ liver regeneration
PC (5 min/10 min) [13]	Immediately before ischemia	↑ PPAR*α*	Obese Zucker rats; warm ischemia (60 min)	↓ hepatic injury	Not evaluated
n-3 PUFA (EPA (270 mg/kg) and DHA (180 mg/kg)) [28]	7 days	↑ PPAR*α*	Sprague-Dawley rats; warm ischemia	↓ hepatic injury, inflammation, and oxidative stress	Not evaluated
EPA (2700 mg/d) [41]	1 year	↑ PPAR*α*	NAFLD patients	↓ steatosis, hepatic injury, necroinflammation, and oxidative stress	Not evaluated
n-3 PUFA (1 g/day) [42]	1 year	↑ PPAR*α*	NAFLD patients	↓ steatosis, hepatic injury, and necroinflammation	Not evaluated
n-3 PUFA (2 g/day) [43]	6 months	↑ PPAR*α*	NAFLD patients	↓ steatosis, hepatic injury, necroinflammation, and hepatic injury	Not evaluated
n-3 PUFA (2 g, 3 times daily) [44]	24 weeks	↑ PPAR*α*	NAFLD patients with hyperlipidemia	↓ steatosis and hepatic injury	Not evaluated
Ω-3 FA (5 mL, thrice daily) [45]	24 weeks	↑ PPAR*α*	NAFLD patients with dyslipidemia	↓ steatosis and hepatic injury	Not evaluated
Atorvastatin (20 mg/daily) [45]	24 weeks	↑ PPAR*α*	NAFLD patients with dyslipidemia	↓ steatosis and hepatic injury	Not evaluated
Orlistat (120 mg, thrice daily) [45]	24 weeks	↑ PPAR*α*	NAFLD patients with dyslipidemia	↓ steatosis and hepatic injury	Not evaluated

PPAR*α* knockout					
Strategies	Time	Effect	Experimental model	Steatosis and hepatic injury	Regeneration

PPAR*α*-knockout [23]	—	↓ PPAR*α*	PPAR*α*-null mice Warm ischemia (90 min)	↑ hepatic injury	Not evaluated
PPAR*α*-knockout [46]	—	↓ PPAR*α*	PPAR*α*-null mice fed HF diet	↑ hepatic *β*-oxidation	Not evaluated
PPAR*α*-knockout [47]	—	↓ PPAR*α*	PPAR*α*-null mice Partial hepatectomy	Not evaluated	↓ liver regeneration

PPAR*γ*					
PPAR*γ* activator					
Strategies	Time	Effect	Experimental model	Steatosis and hepatic injury	Regeneration

Rosiglitazone (10 mg/kg) [6]	30 min before ischemia	↑ PPAR*γ*	PPAR*γ* ^±^ mice	↓ hepatic injury	Not evaluated
Rosiglitazone (2.5 *μ*mol/kg/d) [17]	1 week	↑ PPAR*γ*	Ob/ob mice	↓ triglycerides	Not evaluated
Rosiglitazone (3 mg/kg/day) [48]	5 weeks	↑ PPAR*γ*	PPAR*γ* ^fl/fl^ mice fed HFD diet	↑ steatosis	Not evaluated
Rosiglitazone (1 mg/kg/day) [49]	12 weeks	↑ PPAR*γ*	Obese C57BL/6J mice	↑ steatosis	Not evaluated
Rosiglitazone (10 mg/kg) [50]	2 days before surgery	↑ PPAR*γ*	Mice partial hepatectomy	Not evaluated	↓ hepatic regeneration
Troglitazone (0.1%) + adPPAR*γ* [51]	adPPAR*γ* (5th day) troglitazone (5 days)	↑ PPAR*γ*	PPAR*α*-null mice fed CD diet	↑ steatosis	Not evaluated
Pioglitazone (500 *μ*g/Kg) [52]	8 weeks	↑ PPAR*γ*	Rat fed liquid diet + alcohol	↓ liver injury	Not evaluated
Pioglitazone (30 mg) [53]	96 weeks	↑ PPAR*γ*	Patients with NASH	↓ steatosis	Not evaluated
Pioglitazone (25 mg/kg/day) [54]	5 days before surgery	↑ PPAR*γ*	KK-A^Y^, mice partial hepatectomy	Not evaluated	↑ hepatic regeneration
Pioglitazone (20 mg/kg) [7]	1.5 h before ischemia	↑ PPAR*γ*	Mice Warm ischemia (60 min)	↓ hepatic injury	Not evaluated
Ang II blockers Captopril (100 mg/kg) or PD123319 (30 mg/kg) [36]	Immediately before ischemia	↑ PPAR*γ*	Obese Zucker rats; warm ischemia (60 min)	↓ hepatic injury	Not evaluated
EGF and IGF-1 (10 *μ*g/L) [34]	24 h in UW solution	↑ PPAR*γ*	Obese Zucker rats; isolated liver perfused (24 h cold ischemia)	↓ hepatic injury	Not evaluated
EGF (100 *μ*g/Kg) [35]	3 doses (every 8 h) starting before surgery	↑ PPAR*γ*	Obese Zucker rats; warm ischemia (60 min)	↓ hepatic injury	Not evaluated
IGF-I (400 *μ*g/Kg) [35]	2 doses (every 12 h) starting before surgery	↑ PPAR*γ*	Obese Zucker rats; warm ischemia (60 min)	↓ hepatic injury	Not evaluated
Adenovirus PPAR*γ* + rosiglitazone (50 mg/kg/day) [55]	8 weeks	↑ PPAR*γ*	C57BL/6J mice fed MCD diet	↓ steatohepatitis and fibrosis	Not evaluated
PC (5 min/10 min) [36]	Immediately before ischemia	↑ PPAR*γ*	Obese Zucker rats; warm ischemia (60 min)	↓ hepatic injury	Not evaluated

PPAR*γ* inhibitor					
Strategy	Time	Effect	Experimental model	Steatosis and hepatic injury	Regeneration

GW9662 (1 mg/kg) [14]	1 h before surgery	↓ PPAR*γ*	Liver transplantation (6 h cold ischemia)	Does not change in hepatic injury	Not evaluated
GW9662 (1 mg/kg) [14]	1 h before surgery	↓ PPAR*γ*	Steatotic liver transplantation (6 h cold ischemia)	↓ hepatic injury	Not evaluated
GW9662 (1 mg/kg, 3 times/week) [55]	8 weeks	↓ PPAR*γ*	C57BL/6J mice fed MCD diet	↑ steatohepatitis, fibrosis and hepatic injury	Not evaluated
RBP4 (150 *μ*g/kg) [14]	30 min before surgery	↓ PPAR*γ*	Steatotic liver transplantation (6 h cold ischemia)	↓ hepatic injury	Not evaluated
PC (5 min/10 min) [14]	Immediately before ischemia	↓ PPAR*γ*	Steatotic liver transplantation (6 h of cold ischemia)	↓ hepatic injury	Not evaluated

PPAR*γ* inhibitor					
Strategies	Time	Effect	Experimental model	Steatosis and hepatic injury	Regeneration

PPAR*γ*-knockout [56]	—	↓ PPAR*γ*	Liver-specific PPAR*γ*-null mice	↓ steatosis	Not evaluated
